# Targeting metabolic flexibility via angiopoietin-like 4 protein sensitizes metastatic cancer cells to chemotherapy drugs

**DOI:** 10.1186/s12943-018-0904-z

**Published:** 2018-10-20

**Authors:** Maegan Miang Kee Lim, Jonathan Wei Kiat Wee, Jen Chi Soong, Damien Chua, Wei Ren Tan, Marco Lizwan, Yinliang Li, Ziqiang Teo, Wilson Wen Bin Goh, Pengcheng Zhu, Nguan Soon Tan

**Affiliations:** 1 School of Biological Sciences, Nanyang Technological University Singapore, 60 Nanyang Drive, Singapore, 637551 Singapore; 2 Lee Kong Chian School of Medicine, Nanyang Technological University Singapore, 50 Nanyang Drive, Singapore, 639798 Singapore; 3 Institute of Molecular Cell Biology, Proteos, Agency for Science Technology & Research, 61 Biopolis Drive, Singapore, 138673 Singapore; 4 KK Research Centre, KK Women’s and Children Hospital, 100 Bukit Timah Road, Singapore, 229899 Singapore

**Keywords:** Epithelial-mesenchymal transition, Multi-drug resistance, Angiopoietin-like 4, ATP-binding cassette transporters

## Abstract

Overcoming multidrug resistance has always been a major challenge in cancer treatment. Recent evidence suggested epithelial-mesenchymal transition plays a role in MDR, but the mechanism behind this link remains unclear. We found that the expression of multiple ABC transporters was elevated in concordance with an increased drug efflux in cancer cells during EMT. The metastasis-related angiopoietin-like 4 (ANGPTL4) elevates cellular ATP to transcriptionally upregulate ABC transporters expression via the Myc and NF-κB signaling pathways. ANGPTL4 deficiency reduced IC_50_ of anti-tumor drugs and enhanced apoptosis of cancer cells. In vivo suppression of ANGPTL4 led to higher accumulation of cisplatin-DNA adducts in primary and metastasized tumors, and a reduced metastatic tumor load. ANGPTL4 empowered cancer cells metabolic flexibility during EMT, securing ample cellular energy that fuels multiple ABC transporters to confer EMT-mediated chemoresistance. It suggests that metabolic strategies aimed at suppressing ABC transporters along with energy deprivation of EMT cancer cells may overcome drug resistance.

**Electronic supplementary material:**

The online version of this article (10.1186/s12943-018-0904-z) contains supplementary material, which is available to authorized users.

## Main text

Cytotoxic chemotherapy is one of the mainstays of cancer treatment. Despite being an important therapeutic option for most cancer patients, the development of multiple drug resistance (MDR) by tumors has emerged as a major obstacle that limits the efficacy of chemotherapy [1]. Recent evidence also indicates that epithelial-mesenchymal transition (EMT) plays a role in MDR [2, 3]. Although these studies have oversimplified the relationship between EMT and MDR, they highlight a need for a better understanding of these two complex and poorly understood processes which often co-exist clinically.

A well-established cause of MDR is the increased expression of ATP-binding cassette (ABC) transporters, that efflux various chemotherapeutic compounds from cells [4]. Their broad specificity has been the subject of numerous attempts. However, the results of clinical trials have been rather disappointing. The failure may be attributed to the lack of specificity, resulting in toxicity and adverse drug interaction, or singularly targeting one transporter. Increased expression of ABC transporters necessitates a concomitant increase in cellular adenylate energy to fuel their activities, otherwise the cancer cells will experience an ‘ATP debt.’ Thus, targeting cancer metabolism has emerged as a promising strategy. However, the metabolic flexibility shown by cancer cells during EMT poses significant therapeutic challenges. In this context, the role of angiopoietin-like 4 (ANGPTL4) as a driver of EMT-enriched metabolic changes is a prime target. Numerous clinical and molecular evidence have identified ANGPTL4 as a pro-metastatic gene [5, 6]. Recent studies showed that ANGPTL4 augmented cellular metabolic activity and coordinated the energy demands required for EMT competency [7, 8].

In this study, we explore the attenuation of metabolic flexibility as a potential strategy to attenuate the activities of ABC transporters and to overcome MDR in metastatic cancer cells.

## Results and discussion

### ANGPTL4 elevates cellular ATP to fuel ABC transporters in cancer cells during EMT

We examine the expression of ABC transporters in three in vitro EMT models using the polarized gastric carcinoma line MKN74 [7]. EMT was initiated by either hypoxia (1% O_2_) or TGFβ1. EMT was initiated in MKN74_Snai1ER_, a MKN74 line carrying a Snai1-ER transgene, by 4-hydroxytamoxifen (4-OHT). Upon exposure to stimuli, the MKN74 cells underwent EMT after 48–96 h as confirmed by immunoblotting and qPCR of epithelial- and mesenchymal-associated genes (Additional file 1: Figure S1A-C).

**Fig. 1:**
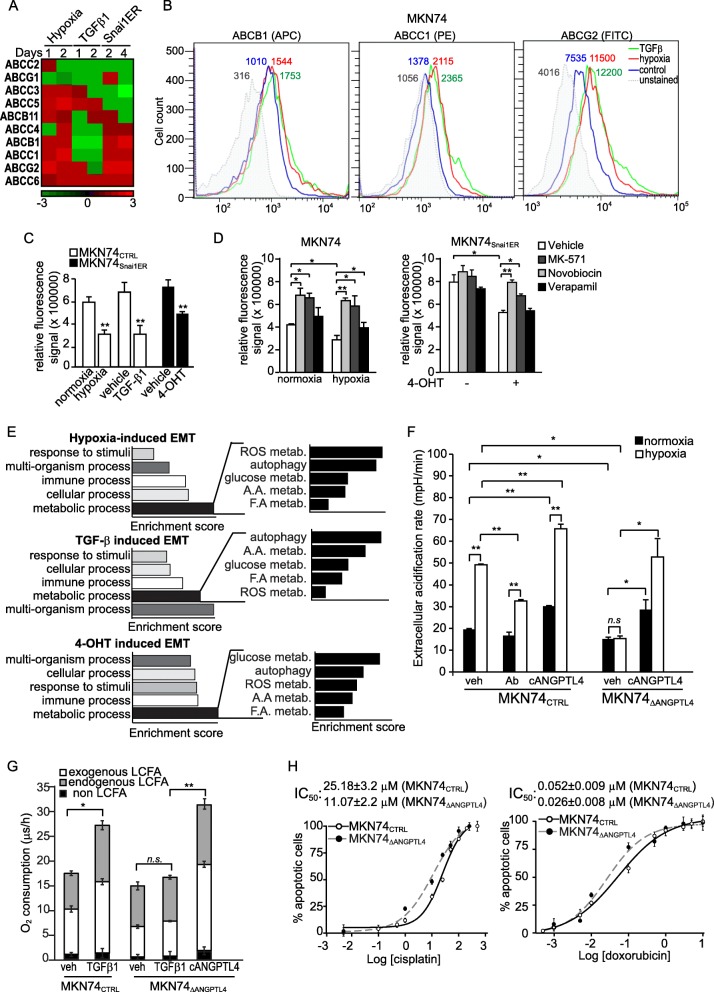
ANGPTL4 increases energy charge to fuel ABC transporters activity. **a** Heatmap showing fold change in the mRNA expression of multiple ABC transporters in three in vitro EMT models. *n* = 3 independent experiments. **b** FACS analysis of cell surface expression of indicated ABC transporters, ABCB1 (left panel), ABCC1 (middle panel) and ABCG2 (right panel) in hypoxia- and TGF-β1-induced EMT of MKN74 cells. Data are represented as mean ± s.d. from at least five independent experiments. **c-d** Relative fluorescence signal of efflux assay measuring drug efflux capacity of MKN74 and MKN74_Snai1ER_ cells in all three in vitro EMT models (**c**), and in the presence of selective ABC transporters inhibitors Verapamil, MK-571 and Novobiocin (**d**). Data are represented as mean ± s.d. from at least five independent experiments. *P < 0.05, **P < 0.01. **e** Gene enrichment analysis based on gene expression data of three in vitro EMT models (GSE71280). **f-g** Extracellular acidification rate (**f**) and long chain fatty acid (LCFA) β-oxidation (**g**) analyses of control (MKN74_CTRL_) and siRNA ANGPTL4-knockdown (MKN74_ΔANGPTL4_) after indicated treatments. Values were normalized to total cellular protein. Values are represented as mean ± s.d. from at least three independent experiments. **h** Graphs showing the IC_50_ curve of MKN74_CTRL_ (solid line) and MKN74_ΔANGPTL4_ (dotted line) to cisplatin (left panel) and doxorubicin (right panel). Values are represented as mean ± s.d. from *n* = 5 independent experiments. *P < 0.05, ** *P* < 0.01

Our focussed gene expression analysis revealed an enrichment of multiple ABC transporters genes, including ABCB1 (MDR1), ABCC1 (MRP1) and ABCG2 (BCRP), across the EMT models (Fig. 1a). Flow cytometry confirmed elevated expression of several ABC transporters during EMT of MKN74 and MCF-7 cancer cells (Fig. 1b and Additional file 1: Figure S1D). Regardless of the stimuli, cancer cells undergoing EMT showed a higher drug efflux capacity as evidenced by a 30–50% decrease in intracellular fluorescent dye (Fig. 1c). Next, the relative contribution of ABCB1, ABCC1 and ABCG2 were determined by using inhibitor Verapamil, MK-571, and Novobiocin, respectively. Our finding highlighted the significance of ABCC1 and ABCG2 in MDR during EMT (Fig. 1d).

ANGPTL4 is a critical player that coordinate cellular metabolic activities that enhances metastasis [7]. We observed a higher ABCB1 protein during EMT of multiple cancer cell lines and stage-specific human tumor biopsies that were positively correlated with cANGPTL4, the C-terminal fragment of ANGPTL4 (Additional file 1: Figure S2A-C). Interrogation of the microarray data (GSE71280), revealed an enrichment of genes regulating glucose, fatty acids and amino acid metabolisms, autophagy and ROS metabolism (Fig. 1e). The suppression of ANGPTL4 resulted in significant alteration of > 50% of genes identified in our microarray analysis, suggesting that ANGPTL4 plays a pivotal role on metabolic flexibility during EMT (Additional file 1: Figure S3A). Consistent with the microarray analysis, the immunoneutralization (α-cANGPTL4) or siRNA knockdown (ΔANGPTL4) of ANGPTL4 reduced 2-NBDG uptake, a fluorescent glucose analog (Additional file 1: Figure S3B) and diminished glycolysis (Fig. 1f). Similar observation was obtained using the MKN74_Snai1ER:shANGPTL4_ cells (Additional file 1: Figure S3C), MKN74_Snai1ER_ harboring doxycycline (dox)-inducible shANGPTL4 transgene. Conversely, recombinant cANGPTL4 (rh-cANGPTL4) treatment increased glycolysis (Fig. 1f). The β-oxidation of long-chain fatty acids was also elevated during TGFβ1-induced EMT, which was abolished by ANGPTL4-knockdown and restored by exogenous rh-cANGPTL4 (Fig. 1g). Furthermore, the cellular ATP concentration was elevated during EMT and reduced upon ANGPTL4 depletion (Additional file 1: Figure S3D-E).

Drug efflux by ABC transporters is an ATP-dependent process. We observed higher fluorescent dye was trapped intracellularly and less actively pumped out in ANGPTL4-depleted cells (Additional file 1: Figure S3F-G). Notably, the IC_50_ of cisplatin and doxorubicin was reduced by ~ 50% in MKN74_ΔANGPTL4_ than MKN74_CTRL_ (Fig. 1h) Altogether, ANGPTL4-mediated metabolic flexibility helps cancer cells secure cellular ATP for EMT-dependent drug efflux by ABC transporters.

### ANGPTL4 deficiency in metastatic cancer cells impairs cisplatin efflux in vivo

**Fig. 2:**
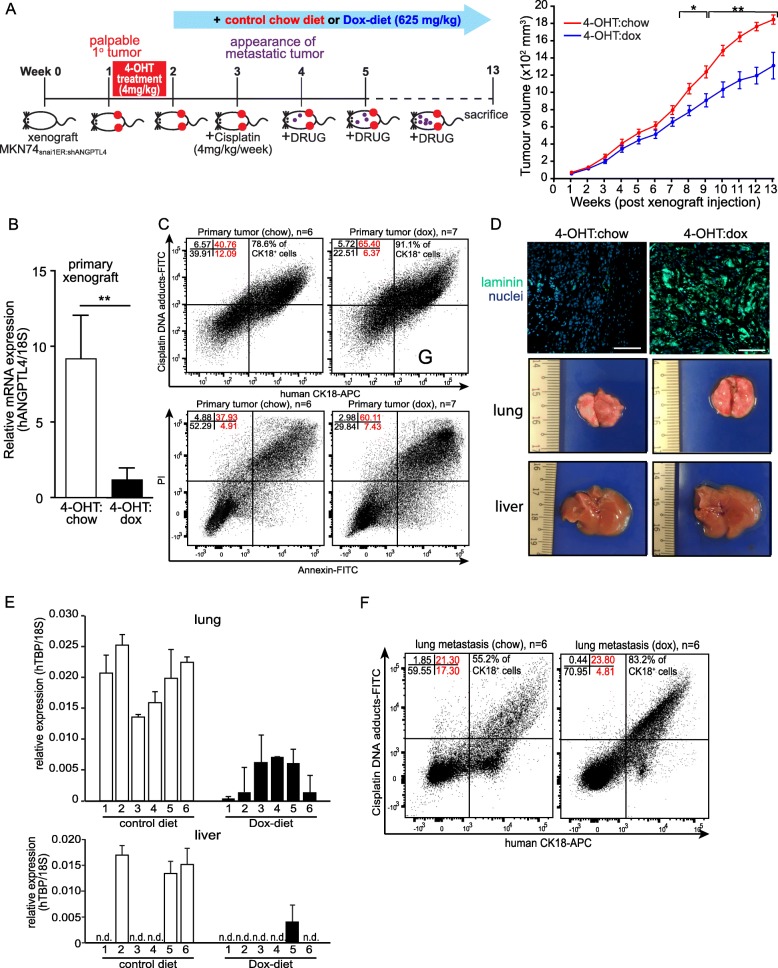
Reduction in ANGPTL4 impairs cisplatin efflux and increases in vivo tumor chemosensitivity. **a** A schematic illustration showing the metastatic xenograft model. MKN74_Snai1ER:shANGPTL4_ cells were injected to form primary xenograft tumors (*n* = 6 per experimental subgroup, two tumors per mouse). All mice were injected twice with 4-OHT to induce EMT of the MKN74_Snai1ER:shANGPTL4_ and treated with cisplatin. Mice were fed with either normal chow diet (4-OHT:chow) or doxycycline containing diet (4-OHT:dox; 625 mg/kg). Cisplatin treatment (4 mg/kg/week) was administered from week 3 (left panel). Primary xenograft tumors were measured with a pair of Vernier calipers with tumor volume set to be (Length×Width×Width)/2 (right panel). **b-c** Relative mRNA expression of ANGPTL4 (**b**) and FACS analysis (**c**) of cisplatin-DNA adduct-positive among CK18^+^ cells (top panel) and apoptotic (Annexin V-PI positive; bottom panel) cells from 4-OHT:chow and 4-OHT:dox primary xenograft tumors. For real-time PCR, ribosomal 18S (18S) was used as a housekeeping gene. Values are mean ± s.d. from *n* = 3 independent experiments with 5–6 mice per time point. **d** Immunofluorescence staining for laminin 332 (green) of 4-OHT:chow and 4-OHT:dox primary xenograft tumors. The nuclei were counterstained with DAPI (blue). Scale bar: 100 μm (top panel). Macroscopic images of lung and liver from mice bearing 4-OHT:chow and 4-OHT:dox primary xenograft tumors. **e** Relative mRNA expression of human TBP in the lung (top panel) and liver (bottom panel) tissues of cisplatin-treated xenograft mice. Human-specific TBP primers were used to identify and quantify human. Ribosomal 18S primers were used to detect both human and mouse cells for normalization. Values are mean ± s.d. of triplicate runs from 6 mice. **f** FACS analysis cisplatin-adduct^+^ cells among human CK18^+^ MKN74 cells that metastasized to the lung tissues of 4-OHT:chow and 4-OHT:dox mice. Analysis showed 38.6% (red) CK18-positive cells in 4-OHT:chow lungs compared with 28.61% (red) 4-OHT:dox lungs. Data are represented as mean ± s.d. from *n* = 3 independent experiments. *P < 0.05, ** *P* < 0.01

To confirm the above observations in vivo, we examined the metastasis of MKN74_Snai1ER:shANGPTL4_ cells in response to cisplatin in the presence or absence of ANGPTL4 (Fig. 2a). Dox-diet (4-OHT:dox) suppressed ANGPTL4 and attenuated primary xenograft growth compared with chow-diet (4-OHT:chow)-derived xenografts (Fig. 2a-b). In 4-OHT:dox xenografts, ~ 91% of cytokeratin18 (CK18)-positive cancer cells has cisplatin-DNA adducts compared with ~ 78% in 4-OHT:chow xenografts (Fig. 2c). Importantly, 4-OHT:dox xenografts have higher apoptotic cancer cells than 4-OHT:chow xenografts (67.54% vs. 42.84%) (Fig. 2c). The 4-OHT:dox tumors showed clear and punctuated laminin332 staining compared with 4-OHT:chow tumors (Fig. 2d). The 4-OHT:dox mice also harbored fewer lung and liver metastases than 4-OHT:chow mice (Fig. 2e-f). Furthermore, ~ 83.2% metastasized CK18-positive cells have cisplatin-DNA adducts in 4-OHT:chow mice compared with 55.2% in 4-OHT:chow mice (Fig. 2f). Altogether, the in vivo suppression of ANGPTL4 led to the higher accumulation of cisplatin-DNA adducts in metastasized tumors, reducing metastatic tumor load.

### Coordinated upregulation of multiple ABC transporters by c-Myc and NF-κB

**Fig. 3:**
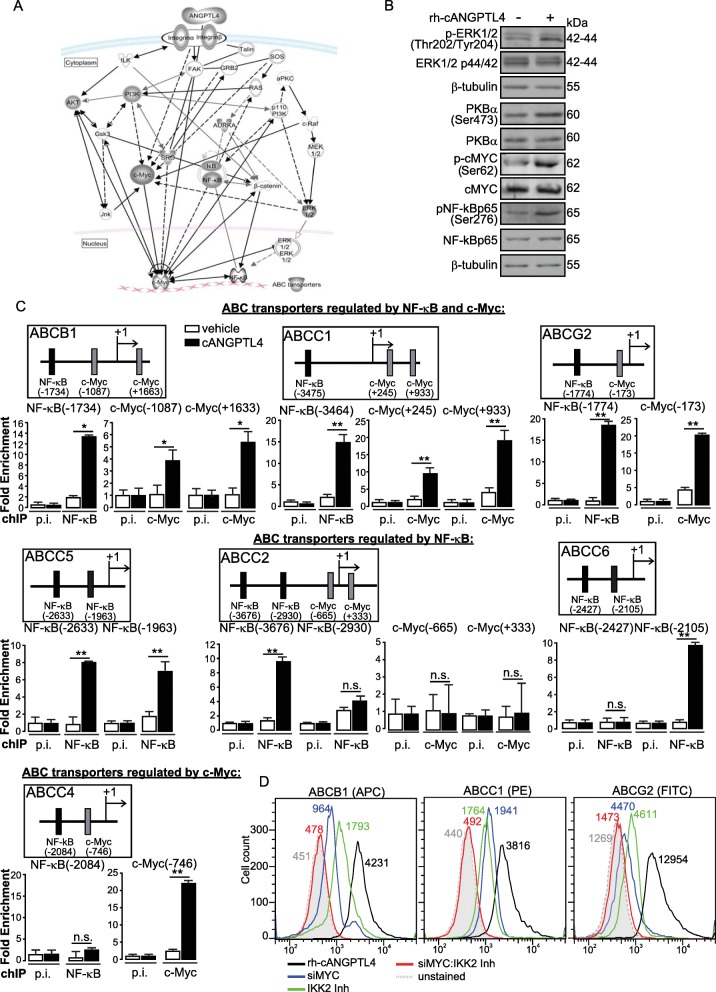
Coordinated transcriptional regulation of multiple ABC transporter genes. **a** Ingenuity Pathway Analysis (IPA) identified c-Myc and NF-κB as potential transcriptional factors that regulate the expression of multiple ABC transporters when stimulated with recombinant cANGPTL4 protein. IPA-assisted pathways were mapped following experimental kinase inhibitor screen. To avoid bias, all kinases that prevented the up-regulation of ABC transporter expression under the stimulation of cANGPTL4 were analyzed using the Ingenuity Pathway Analysis software. **b** Representative immunoblot of total and phosphorylated ERK1/2, PKB (Akt), c-Myc and NF-κB from MKN74 cells treated with recombinant cANGPTL4. β-tubulin serves as loading and transfer controls. Loading controls for the immunoblot analyses were from the same sample. **c** Representative quantitative ChIP assays performed using preimmune IgG (p.i.) or antibodies against phosphorylated NF-κB/p65 and c-Myc in rh-cANGPTL4-treated MKN74 cells. Schematic illustrations show the relative positions of putative NF-κB and Myc binding sites on the regulatory regions of indicated human ABC transporter genes. Putative specific binding sites for respective transcription factors were determined in silico with the Jaspar database. A control region 2 kb upstream of the promoter served as a negative control. Values are represented as mean ± s.d. from *n* = 3 independent experiments. *P < 0.05, ** *P* < 0.01. n.s., not significant. **d** FACS analysis of cell surface expression of ABCB1 (left panel), ABCC1 (middle panel) and ABCG2 (right panel) in recombinant cANGPTL4 (rh-cANGPTL4)-treated MKN74 cells whose c-Myc was suppressed by siRNA (siMYC) and/or NF-κB activity was inhibited by IKK2 inhibitor (IKK2 Inh; 1 μM). Data are represented as mean ± s.d. from 3 independent experiments

The rh-cANGPTL4 upregulated the mRNA levels of seven ABC transporters (Additional file 1: Figure S4A). Following a kinase inhibitor array screen and IPA analysis (Additional file 1: Figure S4B-C), we deciphered that cANGPTL4 activated key signaling mediators of the PI3K/AKT, NF-κB and MEK/ERK pathways and culminated in c-Myc and NF-κB activation (Fig. 3a). Our immunoblot results confirmed the phospho-activation of these kinases and transcription factors in response to rh-cANGPTL4 stimulation (Fig. 3b). In silico analysis of the regulatory promoter regions of these seven ABC transporters [9] identified many putative c-Myc and NF-κB transcription factor binding sites (Fig. 3c, Additional file 1: Table S1). Further investigation showed that rh-cANGPTL4 upregulated ABC transporters which were attenuated by siMYC or IKK2 Inhibitor IV (Fig. 3d and Additional file 1: Figure S4A). Flow cytometry confirmed the elevated expression of ABCB1, ABCC1 and ABCG2 in rh-cANGPTL4-treated MKN74, whose levels were reduced when c-Myc and NF-κB activities were impaired (Fig. 3d). Quantitative chIP analysis on MKN74 treated with rh-cANGPTL4 revealed differential transcriptional regulation of the ABC transporters genes by c-Myc and NF-κB activated by rh-cANGPTL4 (Fig. 3c). The specificity and efficiency of PCR were verified by melt curve analysis (Additional file 1: Figure S5).

We probe the TCGA database to reveal the potential clinical relevance of ANGPTL4 (Additional file 1: Figure S6). Twenty one different tissue sites out of 61 primary sites in TCGA, i.e. 34% of human tumor types, showed alteration in ANGPTL4, with higher representation in uterus, skin and bladder cancers (Additional file 1: Figure S6A). ANGPTL4 has some predictive value in survival prediction due to its high correlation with c-Myc and NF-κB (Additional file 1: Figure S6B-C). Many other factors can explain the survivability of these cases, among which is the post-translational processing of ANGPTL4 to cANGPTL4, which is responsible for the pro-oncogenic action of ANGPTL4.

In summary, our research showed that cancer cells undergoing EMT exhibited metabolic flexibility that secure ample cellular adenylate energy to fuel the increased activities of ABC transporters. Importantly, such re-wiring of metabolic dependency circumvents metabolic therapies designed to target individual metabolic pathways [10]. Our findings underscore the usefulness of metabolic strategies aimed at suppressing ABC transporters along with energy deprivation of EMT cancer cells can overcome drug resistance in metastatic cancer cells. Additionally, the reduction in chemoresistance when anti-ANGPTL4 is used concurrently with conventional chemotherapy agents has the potential to prolong the efficacy of conventional chemotherapy.

## Conclusions

We reveal the relationship between EMT-associated metabolic re-wiring and MDR involves metastasis-related gene ANGPTL4, a driver of the EMT-enriched metabolic programme. ANGPTL4 up-regulates multiple ABC transporters expression in cancer cells during EMT via the activation of NF-κB and c-Myc transcription factors. ANGPTL4 deficiency inhibits metabolic flexibility and deter the development of EMT-mediated chemoresistance in metastatic tumor against cisplatin in vivo.

## Additional file

Additional file 1: Supplementary Information. (PDF 1910 kb)

## Abbreviations

4-OHT: 4-hydroxytamoxifen
ABC: ATP-binding cassette
ANGPTL4: Angiopoietin-like 4
CK18: Cytokeratin 18
Dox: Doxycycline diet
EMT: Epithelial-mesenchymal transition
MDR: Multidrug resistance
qChIP: Quantitative chromatin immunoprecipitation
qPCR: Quantitative real-time PCR
rh-cANGPTL4: Recombinant human cANGPTL4
α-cANGPTL4: Antibody against cANGPTL4
ΔANGPTL4: siRNA knockdown against ANGPTL4
