# Supplementary material for: Targeting metabolic flexibility via angiopoietin-like 4 protein sensitizes metastatic cancer cells to chemotherapy drugs
Source: Mol Cancer. 2018 Oct 20;17:152. doi: 10.1186/s12943-018-0904-z (PMC6195749; doi:10.1186/s12943-018-0904-z)
Supplement: Supplementary file 1 — Supplementary Information. (PDF 1910 kb) [file 12943_2018_904_MOESM1_ESM.pdf]

Targeting metabolic flexibility via angiopoietin-like 4 protein sensitizes metastatic cancer cells to chemotherapy drugs.

Maegan Miang Kee LIM[1], Jonathon Wei Kiat WEE[1], Jen Chi SOONG[1], Damien CHUA[1], Wei Ren TAN[1], Marco LIZWAN[1], Yinliang LI[1], Ziqiang TEO[1], Wilson Wen Bin GOH[1], Pengcheng ZHU[1], Nguan Soon TAN[1,2,3,4].

[1]School of Biological Sciences, Nanyang Technological University Singapore, 60 Nanyang Drive, Singapore 637551
[2]Lee Kong Chian School of Medicine, Nanyang Technological University Singapore, 50 Nanyang Drive, Singapore 639798
[3]Institute of Molecular Cell Biology, 61 Biopolis Drive, Proteos, Agency for Science Technology & Research, Singapore 138673
[4]KK Research Centre, KK Women's and Children Hospital, 100 Bukit Timah Road, Singapore 229899

Emails:
Maegan Miang Kee LIM (MLIM022@e.ntu.edu.sg), Jonathan Wei Kiat WEE (wkwee@ntu.edu.sg), Jen Chi SOONG (JSOONG003@e.ntu.edu.sg), Damien CHUA (DChua019@e.ntu.edu.sg), Wei Ren TAN (WTan074@e.ntu.edu.sg), Marco LIZWAN (MLizwan001@e.ntu.edu.sg), Yinliang LI (YLI033@e.ntu.edu.sg), Ziqiang TEO (brian.teo@takeda.com), Wilson Wen Bin GOH (wilsongoh@ntu.edu.sg), Pengcheng ZHU (PCZhu@ntu.edu.sg), Nguan Soon TAN (nstan@ntu.edu.sg).

Supplementary Information

Materials and Methods

Antibodies and reagents. Otherwise indicated, all antibodies were purchased from Cell Signaling, USA. IRdye 680-conjugated secondary antibodies were purchased from Thermo Scientific, USA. Antibodies against Cisplatin modified DNA antibody [CP9/19] and Laminin 332 antibody (ab14509) were from abcam. 4-hydroxytamoxifen (4-OHT), cisplatin and doxycycline (Dox) were purchased from Sigma-Aldrich, USA. Recombinant human cANGPTL4 (rh-cANGPTL4) were expressed and purified in-house as previously described [1, 2]. Neutralizing cANGPTL4 antibodies (α-cANGPTL4) and its specificity were previously described [3, 4]. ON-TARGETplus siRNA against ANGPTL4 (ΔANGPTL4) was purchased from GE Dharmacon (USA).

Cell culture and EMT induction. Human gastric carcinoma line MKN74 (ATCC, USA) are maintained in RPMI-1640 (GE Healthcare, USA) supplemented with 10% Fetal Bovine Serum in a humidified incubator kept at 5% CO_2 at 37 ºC. The MKN74 cell line which possesses the Snai1-ER transgene ($MKN74_{Snai1ER}$) and $MKN74_{Snai1ER:shANGPTL4}$ cell lines harboring the doxycycline (Dox)-inducible pSingle-tTs-shRNA vector (shANGPTL4 or scrambled shRNA) in $MKN74_{Snai1ER}$ cells were created as previously described [5]. $MKN74_{Snai1ER}$ colonies were induced into EMT upon exposure to 20 ng/mL 4-OHT for 4 days. In hypoxia treatments, the O_2 concentration was reduced to 1% during EMT induction in the hypoxic chamber (Stem Cell Technology, USA) for 2 days. EMT was also induced in MKN74 cells using 10 ng/mL TGF-β1 for 2 days. Transient siRNA knockdown of ANGPTL4 was performed on MKN74 and $MKN74_{Snai1ER}$ ($MKN74_{\Delta ANGPTL4}$ and $MKN74_{Snai1ER:\ \Delta ANGPTL4}$, respectively) using SMARTpool ON-TARGETplus siRNA in accordance with the manufacturer's recommendations. DharmaFECT 1 transfection reagent

was used (GE Healthcare Dharmacon, USA). SMARTpool ON-TARGETplus siRNA is a mixture of 4 siRNA provided as a single reagent.

Human tumor biopsies. Human frozen tissue biopsies from head and neck, breast, gastric and colon were purchased from Proteogenex, USA (**Table S2**).

Protein Extraction and Immunoblot Analysis. Proteins were isolated in cold M-Per Mammalian Protein Extraction Reagent (Thermo Scientific) and normalized using the NanoDrop 3300 Spectrophotometer (Thermo Scientific). Protein bands were resolved using SDS-PAGE gels and later transferred onto low fluorescence PVDF membranes (Merck Millipore, USA). All loading controls for immunoblot analyses were obtained from the same sample. Immunoblot membranes were blocked using 0.5× Odyssey Blocking Buffer (LI-COR Biotechnology, USA), probed with the respective primary antibodies at 4 °C in 1× Blocking Buffer in TBST [50 mM Tris-HCl, 150 mM NaCl, 0.1% Tween-20, pH 7.6] and later with its respective secondary antibody for 1 h at room temperature, dried and scanned using the CLx scanner and Image Studio V2.1 (LI-COR Biosciences, USA). Loading controls were obtained from the same sample.

RNA Extraction and real-time PCR. Total RNA was extracted using TRIzol (Invitrogen, USA) and was reverse transcribed using iScript Reverse Transcription Supermix according to the manufacturer's protocol (Bio-Rad, USA). Quantitative PCR (qPCR) was performed as previously described. Primer sequences are summarized in **Table S2**.

Glycolysis analysis. Extracellular acidification rate (ECAR) was measured in real-time using a XF96 flux analyzer (Seahorse Bioscience, USA). Cells were seeded on 96-well microplates. All measurements were normalized against total protein content from cell lysates.

Fatty Acid Oxidation Assay. Cells were induced into EMT with TGFβ1 for 2 days and performed fatty acid oxidation assay using the MitoXpress FAO Kit as per manufacturer's protocol (Sapphire North America, USA).

ATP quantification. ATP level in the cancer cells were quantified using the ATP determination assay kit (Invitrogen, USA) as per manufacturer protocols. Briefly, ATP was released from the cells using somatic cell lysis buffer (Sigma Aldrich, USA) after treatment for indicated time. ATP levels were assayed, and the luminescence readings were taken using a GloMax 20/20 luminometer (Promega, USA). Luminescence readings were plotted against an ATP standard curve to obtain the actual ATP concentration followed by normalization to the protein concentration.

Fluorescence efflux assay

The activities of the multidrug resistance proteins MDR1, MRP and BCRP were measured using eFluxx-ID Green Multidrug Resistance Assay Kit according to the manufacturer's instructions (ENZ-51029-K100, Enzo Lifesciences, Farmingdale, NY). Briefly, single cell suspensions were harvested and equal numbers of cells ($2x10^5$/condition) were resuspended in full media containing either DMSO or specific inhibitors (Verapamil: ABCB1 inhibitor; MK-571: ABCC1 inhibitor; Novobiocin: ABCG2 inhibitor; 200 μm) and incubated for 10 min at 37°C. EFLUXX-ID® Green Detection Reagent was then added and incubated at 37 °C for 40 min before FACS analysis using BD Accuri C6 flow cytometer

(BD Biosciences, USA). Data were analyzed using FlowJo and plotted based on mean intensity.

***In vivo* induction of EMT.** Six-week-old male NSG mice (NOD.Cg-Prkdcscid Il2rgtm1Wjl/SzJ) were injected subcutaneously with 1×10^6 $MKN74_{Snai1ER:shANGPTL4}$ cells resuspended in Matrigel. All mice were injected intraperitoneally with 4-OHT (4 mg/kg) after a week. The mice were then randomly divided into two subgroups and fed on either normal chow or Dox diet (625 mg/kg, ENVIGO, Huntingdon, UK). Cisplatin (4 mg/kg/week) treatment were administered to all mice from week 3.

Flow cytometry. Fresh tissue samples were dissociated using the gentleMACS Dissociator (Miltenyi Biotec, Germany) according to the manufacturer's protocol. Tissue homogenates were filtered using a 700 µm nylon cell strainer and washed before incubating with primary antibodies. Flow cytometry was performed using Accuri C6 Flow Cytometer (BD Biosciences, USA) and analyses were completed on FlowJo v10.0.7 (FlowJo LLC, USA).

Tissue processing and immunofluorescence. Fresh tissue samples were harvested, fixed in 4% paraformaldehyde, dehydrated and embedded in paraffin as previously described[5, 6]. Tissue sections were rehydrated and antigen-retrieved before incubation in 3% normal goat serum for 1 h, then incubated with primary antibodies overnight at 4 °C. Subsequently, tissue sections were washed and incubated with secondary Alexa Fluor 488-conjugated antibodies (Invitrogen, USA) and mounted with Hoeschst 33342 dye (Life Technologies, USA).

Kinase inhibitor array. MKN74 cells were treated with 95 different kinase inhibitors (SYN-2103; Synkinase, Australia) in the absence and presence of rh-cANGPTL4 (10 µg/mL) for 6 h.

Total RNA was isolated, and reverse transcribed using the iScript Reverse Transcription Supermix. qPCR was done using the KAPA^{TM} SYBR qPCR Universal Master Mix (KAPABiosystems). The qPCR primer sequences are listed in **Table S3.**

Partek and IPA. Microarray data were analysed using Partek Genomic Suite (v6.6) to generate heat map. Ingenuity Pathway Analysis (IPA) software was used to conduct pathway analysis and map downstream signalling molecules.

Chromatin-immunoprecipitation (ChIP). The qChIP primer sequences were designed based on putative binding sites of c-MYC and NF-κB detected by JASPAR database [7] or from published work [8]. ChIP experiments were carried out as previously described [4, 9]. Sonicated chromatin complexes were immunoprecipitated using antibodies against p65(NF-κB) or p-c-Myc. Immunocomplexes were affinity precipitated by Protein A/G Sepharose (Santa Cruz, USA). The ChIP primer sequences are in **Figure S3**.

Statistical analysis. Statistical analyses were performed using two-tailed Mann-Whitney U tests using the GraphPad Prism software (La Jolla California, USA). A p-value < 0.05 denote statistically significant differences between means.

Supplementary Figure Legends

Figure S1. Three *in vitro* models of EMT

(**A-C**) Relative fold change in mRNA levels of EMT markers (top panel) and immunoblot analysis of E-cadherin and Snai2 expression (bottom panel) in MKN74 cells exposed to hypoxia (1% O_2) (A), TGF-β (B), as well as in $MKN74_{Snai1ER}$ cells (C) treated with 4-hydroxytamoxifen (4-OHT) at indicated time points. $MKN74_{Snai1ER}$ is a transgenic MKN74 cell line carrying a Snai1-ER transgene that allows direct initiation of EMT by 4-OHT. Epithelial-associated genes (E-cadherin, CDH1; discoidin domain receptor 1, DDR1; receptor-tyrosine kinase, ErBb3) and mesenchymal-associated genes (Snai1; zinc finger E-box binding homeobox 1, ZEB-1). TATA-box binding protein (TBP) was used a housekeeping gene for real-time PCR. β-tubulin protein was used a loading and transfer controls for immunoblot analysis. Data are represented as mean±s.d. from at least three independent experiments. Loading controls for the immunoblot analyses were from the same sample.

(D) FACS analysis of cell surface expression of ABCB1 (left panel), ABCC1 (middle panel) and ABCG2 (right panel) in hypoxia- and TGF-β1-induced EMT of MCF-7 cells. Data are represented as mean±s.d. from n=3 independent experiments.

Figure S2. Increased expression of ABC transporters and cANGPTL4 during EMT of different cancer cell lines and at various stages of human cancers.

(A-C) Relative protein level of ABCB1 and cANGPTL4 in hypoxia- and TGFβ1-induced EMT of MKN74 and in 4-OHT-induced EMT of $MKN74_{Snail1ER}$ (A), in TGFβ1-induced EMT of amely skin squamous cell carcinoma HSC and II4, hepatocarcinoma HepG2 and breast cancer MCF7 cells (B), and in various stages of cancer (C). Comparison was made against respective stage I samples. Representative immunoblot pictures and densitometric

quantification plots are shown. Loading controls (β-tubulin or GADPH) for the immunoblot analyses were from the same sample. Data are represented as mean±s.d. from n=3 independent experiments. *P<0.05, ** P<0.01.

Figure S3. Metabolic flexibility in cancer cells undergoing EMT.

(A) Heat map representation of commonly regulated genes across the three EMT models (control vs. ANGPTL4-knockdown) over the indicated time points. Green: down-regulated; Red: up-regulated; White box: groups of genes that are differentially regulated in ANGPTL4-knockdown cells compared to control cells.

(B) FACS analysis of the fluorescent glucose analog 2-NBDG uptake in MDA-MB-231 (breast) and A549 (lung) cell lines treated with or without neutralizing antibody against ANGPTL4 (α-cANGPTL4) or in control (Scrambled) and ANGPTL4-knockdown (ΔANGPTL4) A-5RT3 (skin) and MKN74 (stomach) cell lines (*left panel*). Relative mRNA and protein levels of ANGPTL4 in control (Scrambled) and ANGPTL4-knockdown (ΔANGPTL4) A-5RT3 and MKN74 cells (right panel). Data are represented as mean±s.d. from n=5 independent experiments. *P<0.05, ** P<0.01.

(C) Extracellular acidification rate of $MKN74_{Snai1ER:shANGPTL4}$ cells after indicated treatments. Values were normalised to total cellular protein. Values are represented as mean±s.d. from at least three independent experiments.

(D-G) Cellular ATP concentration (D-E) and relative fluorescent dyes efflux (F-G) in $MKN74_{CTRL}$ and $MKN74_{\Delta ANGPTL4}$ (D, F), and $MKN74_{Snai1ER:shANGPTL4}$ and $MKN74_{Snai1ER}$ cells (E, G) after indicated treatments. ATP concentration was determined from a standard curve using pure ATP. Values were normalised to total cellular protein. Values are represented as mean±s.d. from at least three independent experiments. Veh: vehicle; Blocking antibody against ANGPTL4: α-cANGPTL4.

Figure S4. ANGPTL4 upregulates ABC transporters expression via PI3K/AKT, MEK/ERK, c-Myc and NF-κB.

(A) Relative fold change in mRNA levels of indicated ABC transporters in indicated MKN74 cells after 6 h exposure to recombinant cANGPTL4 protein (rh-cANGPTL4) treatment (left panel). MKN74 cells whose endogenous c-Myc was suppressed by siRNA (siMYC) and/or treated IKK2 Inhibitor IV (IKK2 Inh). We showed that rh-cANGPTL4 upregulated the mRNA level of seven of these ABC transporters. The knockdown efficiency for cMYC was determined by qPCR and western blot (right panel). β-actin as loading control was from the same samples. Data are represented as mean±s.d. from n=3 independent experiments. *P<0.05, ** P<0.01. n.s. not significant.

(B) A schematic illustration of the rationale behind the kinase inhibitor array, which led up to mapping the pathway in Figure 4a. When MKN74 cells were treated with rh-cANGPTL4, ABC transporter gene expression was increased (left panel). Kinase inhibitors (XXn) that negated this up-regulation of ABC transporters in the presence of rh-cANGPTL4 meant that these kinases were involved in the ANGPTL4-mediated regulation of MDR1 expression (middle panel). Conversely, the addition of kinase inhibitors that did not affect the increase in ABC transporters expression in the presence of rh-cANGPTL4 (right panel) suggested that the target kinase was not involved in ANGPTL4-mediated signaling of ABC transporters.

(C) Relative mRNA level of ABCB1 after treatment with various kinase inhibitors from a Kinase Library (#Synkinase-SYN-2103). Values denotes mean fold change in ABCB1 expression from n=3 independent experiment. All qPCR was performed in triplicates. *P<0.05, ** P<0.01, *** P<0.001.

Figure S5. Specificities of quantitative ChIP (qChIP) PCR.

The ChIP primer sequences and the annealing temperature (Tm) used for qChIP of the seven ABC transporters were indicated. The melting curve analysis were performed to check the specificities of the qChIPs

Figure S6. Alteration in ANGPTL4 based on TCGA database.

(A) Frequency distribution for cases with ANGPTL4 alteration across TCGA projects.

(B) Percentage of cases in TCGA database with alterations in NF-κB, c-MYC and ANGPTL4.

(C) Survival curves for cases with ANGPTL4 alteration and those without ANGPTL4 alteration but with NF-κB and c-MYC alteration. Eight cases are dropped from 348 due to lack of survival data.

Table S1. ChIP transcription binding site details

Target Gene	Transcription Factor	Putative Binding Site Location	Putative Binding Site Sequence	Functional (as verified by qChIP)
ABCB1	NF-kB	-1734	AGGGAGACCCCCT	✓
	c-Myc	-1087	CCATGTGACC	✓
	c-Myc	+1663	CATGTG	✓
ABCC1	NF-kB	-3475	TGGGGTTTCCT	✓
	c-Myc	+245	GCCGCGTGAG	✓
	c-Myc	+933	GCCGCGTGCC	✓
ABCC2	NF-kB	-3676	GGGAAATTCTC	✓
	NF-kB	-2930	GGGTATTTTCA	×
	c-Myc	-665	CATGTGCC	×
	c-Myc	+333	GAACAAGTGGT	×
ABCC4	NF-kB	-2084	TGGGGTTTCACCA	×
	c-Myc	-746	CATGTG	✓
ABCC5	NF-kB	-2633	TGGTGAAACCCCG	✓
	NF-kB	-1963	GTGAAACCCC	✓
ABCC6	NF-kB	-2427	AGGGGGTACACCC	×
	NF-kB	-2105	AGGGTGTACCCC	✓
ABCG2	NF-kB	-1774	AGGTGATCCACCT	✓
	c-Myc	-173	GACACGTG	✓

Table S2. Human tumor biopsies details

Sample Source	Sex	Age	Diagnosis	Histological diagnosis	Grade	TNM	Stage
Breast	F	51	cancer	infiltrating ductal carcinoma	G2	T1cN0M0	I
Breast	F	62	cancer	infiltrating lobular carcinoma	G2	T2N3aM0	IIIC
Breast to ovary	F	59	metastases	infiltrating ductal carcinoma	N/A	recurrent	
Colon	M	66	cancer	adenocarcinoma	G1	T2N0M0	I
Colon	M	80	cancer	adenocarcinoma	G1	T3N1M0	IIIB
Colon to lung	F	42	metastases	adenocarcinoma	N/A	recurrent	
Stomach	M	64	cancer	adenocarcinoma	G2	T2N0M0	IB
Stomach	M	58	cancer	adenocarcinoma	G3	T3N2M0	IIIA
Tongue	M	64	cancer	squamous cell carcinoma	G2	T2N1M0	III
Tongue	M	58	cancer	squamous cell carcinoma	G1	T2N0M0	II

Table S3. Real-time qPCR Primers

Genes	Forward Sequence (5’ to 3’)	Reverse Sequence (3’ to 5’)
**18S**	GTAACCCGTTGAACCCCATT	CCATCCAATCGGTAGTAGCG
**ANGPTL4**	TCCAACGCCACCCACTTAC	TGAAGTCATCTCACAGTTGACCA
**CDH1**	GCCGAGAGCTACACGTTCA	GACCGGTGCAATCTTCAAA
**MDR1**	GGTCTTTCCACTAAAGTCGGAGT	TCCCCTTCAAGATCCATCC
**DDR1**	CACTCCGCTCCCTGTGTC	AGAGGTGCGACTGGAACAA
**ERBB3**	CTGATCACCGGCCTCAAT	GGAAGACATTGAGCTTCTCTGG
**Snai1**	GCCTTCAACTGCAAATACTGC	CTTCTTGACATCTGAGTGGGTC
**TBP**	GCTGGTTATCGGGAGTTGG	ACTGGCCTGGTGTCCTAGAG
**hTBP**	GTGGGGAGCTGTGATGTGAA	GGGAGGCAAGGGTACATGAG
**ZEB1**	GCCAACAGACCAGACAGTGTT	TCTTGCCCTTCCTTTTCCTG

References

1. Goh YY, Pal M, Chong HC, Zhu P, Tan MJ, Punugu L, Lam CR, Yau YH, Tan CK, Huang RL, et al: Angiopoietin-like 4 interacts with integrins beta1 and beta5 to modulate keratinocyte migration. Am J Pathol 2010, 177:2791-2803.
2. Goh YY, Pal M, Chong HC, Zhu P, Tan MJ, Punugu L, Tan CK, Huang RL, Sze SK, Tang MB, et al: Angiopoietin-like 4 interacts with matrix proteins to modulate wound healing. J Biol Chem 2010, 285:32999-33009.
3. Zhu P, Tan MJ, Huang RL, Tan CK, Chong HC, Pal M, Lam CR, Boukamp P, Pan JY, Tan SH, et al: Angiopoietin-like 4 protein elevates the prosurvival intracellular O2(-):H2O2 ratio and confers anoikis resistance to tumors. Cancer Cell 2011, 19:401-415.
4. Chong HC, Chan JS, Goh CQ, Gounko NV, Luo B, Wang X, Foo S, Wong MT, Choong C, Kersten S, Tan NS: Angiopoietin-like 4 stimulates STAT3-mediated iNOS expression and enhances angiogenesis to accelerate wound healing in diabetic mice. Mol Ther 2014, 22:1593-1604.
5. Teo Z, Sng MK, Chan JSK, Lim MMK, Li Y, Li L, Phua T, Lee JYH, Tan ZW, Zhu P, Tan NS: Elevation of adenylate energy charge by angiopoietin-like 4 enhances epithelial-mesenchymal transition by inducing 14-3-3gamma expression. Oncogene 2017, 36:6408-6419.
6. Chan JSK, Sng MK, Teo ZQ, Chong HC, Twang JS, Tan NS: Targeting nuclear receptors in cancer-associated fibroblasts as concurrent therapy to inhibit development of chemoresistant tumors. Oncogene 2018, 37:160-173.
7. Khan A, Fornes O, Stigliani A, Gheorghe M, Castro-Mondragon JA, van der Lee R, Bessy A, Cheneby J, Kulkarni SR, Tan G, et al: JASPAR 2018: update of the open-

access database of transcription factor binding profiles and its web framework. Nucleic Acids Res 2018, 46:D260-D266.

8. Porro A, Haber M, Diolaiti D, Iraci N, Henderson M, Gherardi S, Valli E, Munoz MA, Xue C, Flemming C, et al: Direct and coordinate regulation of ATP-binding cassette transporter genes by Myc factors generates specific transcription signatures that significantly affect the chemoresistance phenotype of cancer cells. J Biol Chem 2010, 285:19532-19543.

9. Lam CR, Tan MJ, Tan SH, Tang MB, Cheung PC, Tan NS: TAK1 regulates SCF expression to modulate PKBalpha activity that protects keratinocytes from ROS-induced apoptosis. Cell Death Differ 2011, 18:1120-1129.

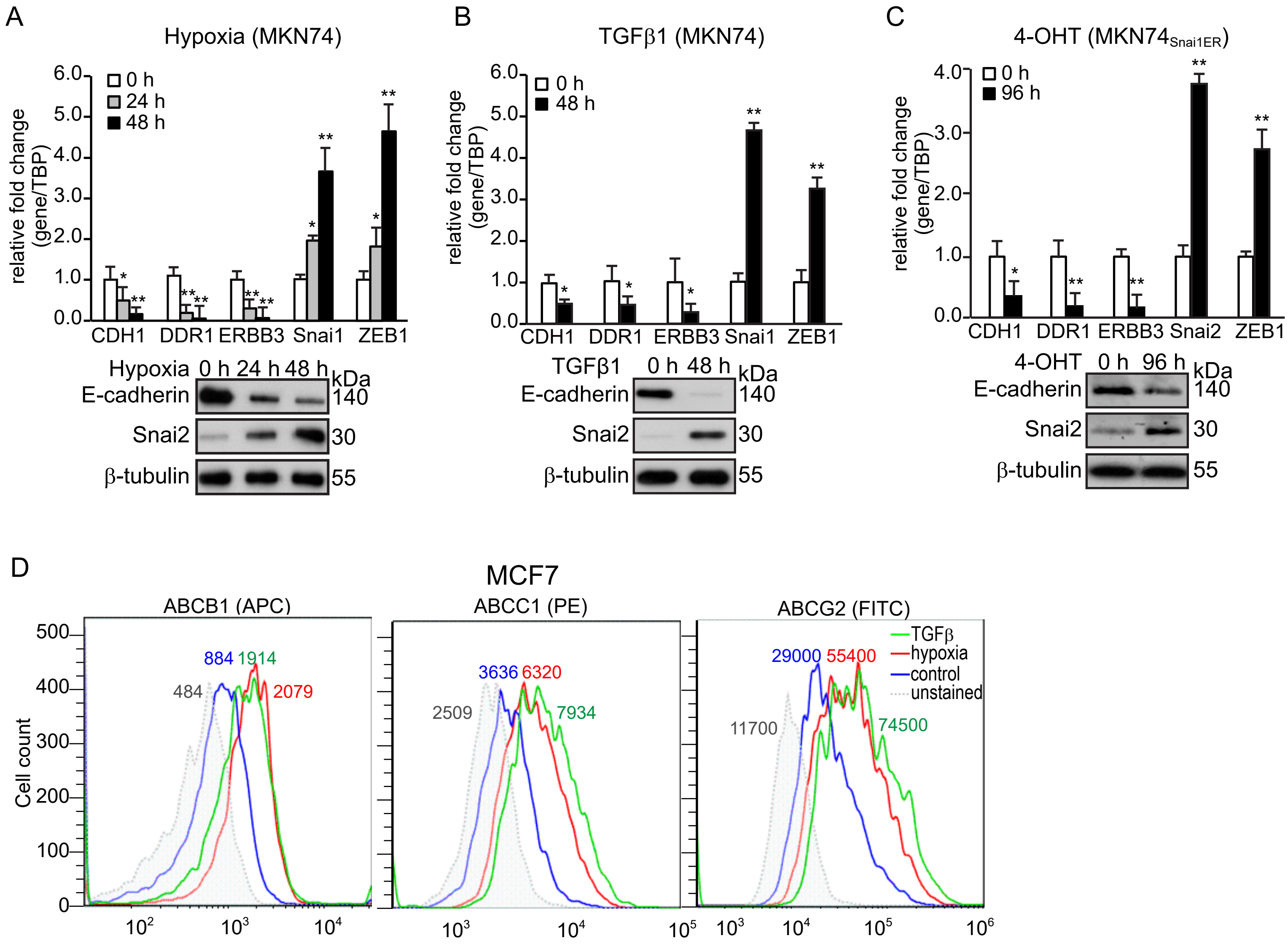
A
Hypoxia (MKN74)
relative fold change (gene/TBP)
0 h
24 h
48 h
CDH1
DDR1
ERBB3
Snai1
ZEB1
Hypoxia 0 h 24 h 48 h kDa
E-cadherin 140
Snai2 30
β-tubulin 55
B
TGFβ1 (MKN74)
0 h
48 h
TGFβ1 0 h 48 h kDa
C
4-OHT (MKN74Snai1ER)
0 h
96 h
4-OHT 0 h 96 h kDa
CDH1
DDR1
ERBB3
Snai2
ZEB1
D
MCF7
ABCB1 (APC)
ABCC1 (PE)
ABCG2 (FITC)
Cell count
884 1914
484
2079
3636 6320
2509
7934
29000 55400
11700
74500
TGFβ
hypoxia
control
unstained

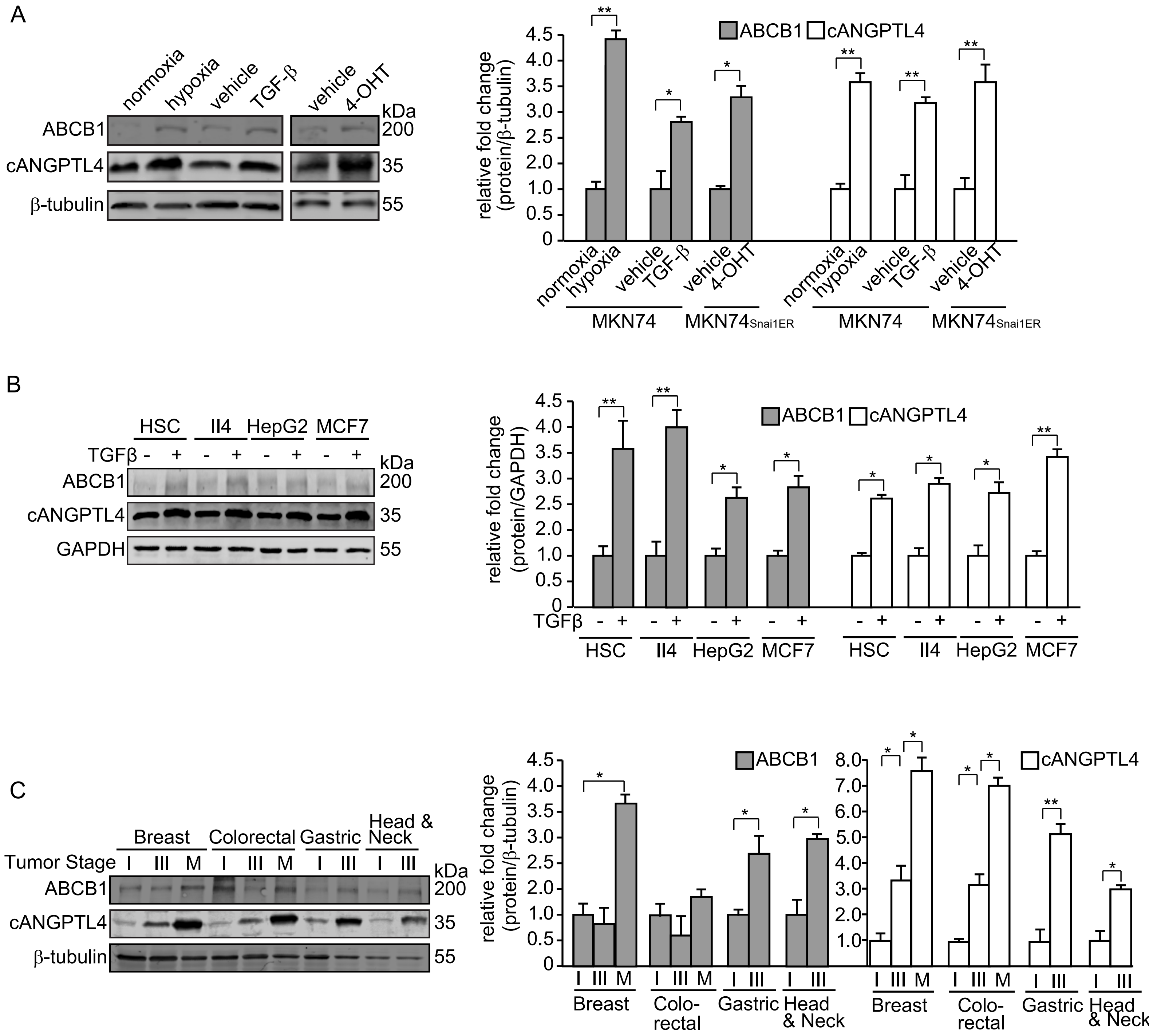
A
normoxia
hypoxia
vehicle
TGF-β
vehicle
4-OHT
kDa
ABCB1 200
cANGPTL4 35
β-tubulin 55
ABCB1
cANGPTL4
relative fold change (protein/β-tubulin)
MKN74
MKN74Snai1ER
B
HSC
Il4
HepG2
MCF7
TGFβ
GAPDH
relative fold change (protein/GAPDH)
C
Breast
Colorectal
Gastric
Head & Neck
Tumor Stage
I
III
M
Colo-rectal

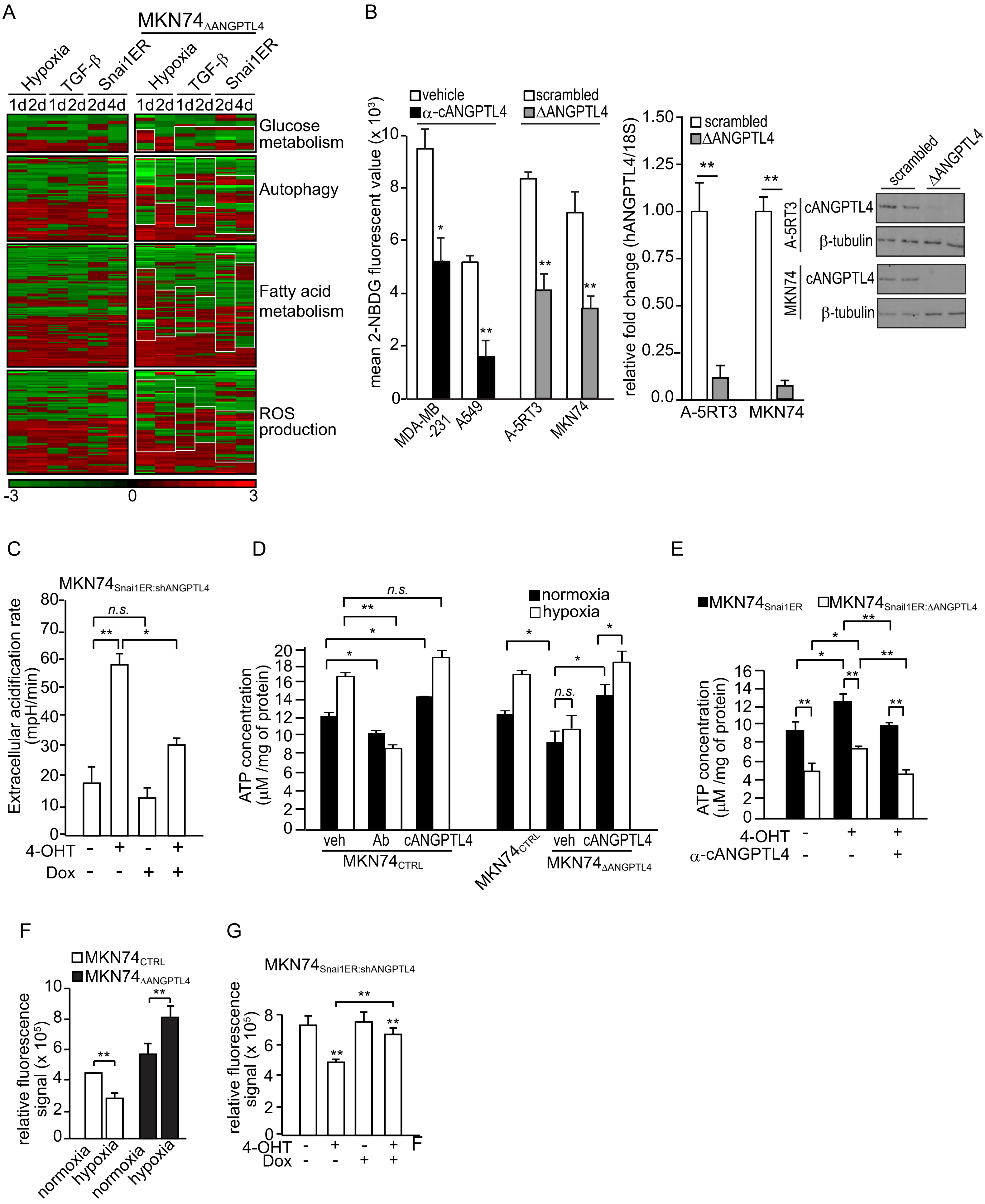
A
MKN74ΔANGPTL4
Hypoxia
TGF-β
Snai1ER
1d 2d 1d 2d 2d 4d
Glucose metabolism
Autophagy
Fatty acid metabolism
ROS production
-3
0
3
B
vehicle
α-cANGPTL4
scrambled
ΔANGPTL4
mean 2-NBDG fluorescent value (x 10³)
MDA-MB-231
A549
A-5RT3
MKN74
relative fold change (hANGPTL4/18S)
cANGPTL4
β-tubulin
C
MKN74Snai1ER:shANGPTL4
Extracellular acidification rate (mpH/min)
n.s.
4-OHT
Dox
D
normoxia
hypoxia
n.s.
ATP concentration (µM /mg of protein)
veh
Ab
cANGPTL4
MKN74CTRL
MKN74ΔANGPTL4
E
MKN74Snai1ER
MKN74Snai1ER:ΔANGPTL4
α-cANGPTL4
F
relative fluorescence signal (x 10⁵)
G

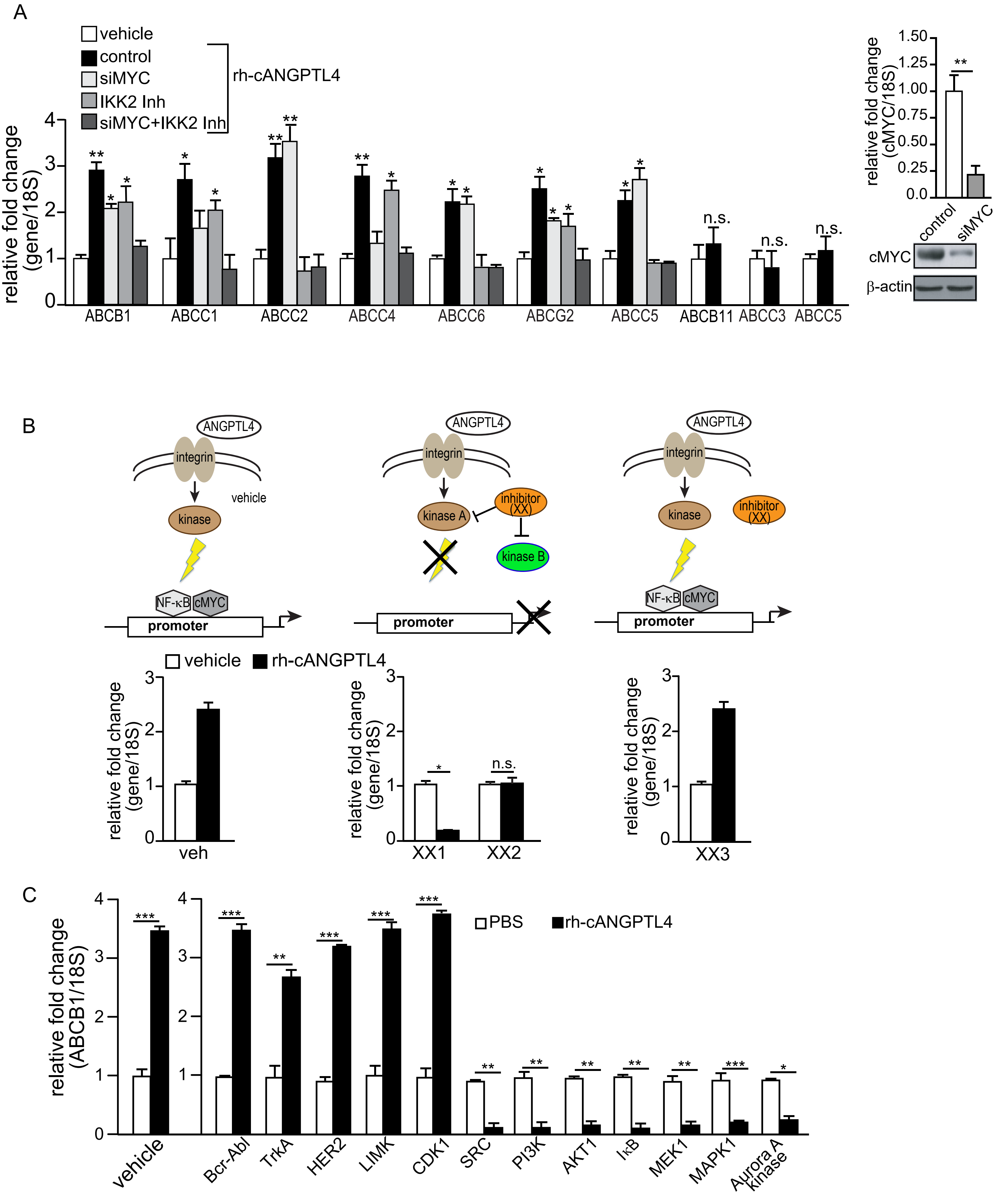
A
vehicle
control
siMYC
IKK2 Inh
siMYC+IKK2 Inh
rh-cANGPTL4
relative fold change (gene/18S)
ABCB1
ABCC1
ABCC2
ABCC4
ABCC6
ABCG2
ABCC5
ABCB11
ABCC3
ABCC5
n.s.
relative fold change (cMYC/18S)
control
siMYC
cMYC
β-actin
B
ANGPTL4
integrin
vehicle
kinase
NF-κB
cMYC
promoter
kinase A
inhibitor (XX)
kinase B
vehicle
rh-cANGPTL4
relative fold change (gene/18S)
veh
XX1
XX2
XX3
n.s.
C
relative fold change (ABCB1/18S)
PBS
rh-cANGPTL4
vehicle
Bcr-Abl
TrkA
HER2
LIMK
CDK1
SRC
PI3K
AKT1
IκB
MEK1
MAPK1
Aurora A kinase

ABCB1

Melt peak

NF-κB

Fw 5'-CCTGTGGCTTCACTGGAGTT-3'
Rv 5'- GTCCAACCTGGCATTGTCTT-3'
Tm 63 °C

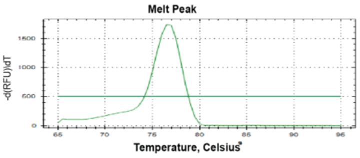

c-Myc A

Fw 5'-TCTCGTCTAACCAGTTGTCTATCTTC-3'
Rv 5'-TACCAGCATTTATTATTAAGTTTAGTG AGG-3'
Tm 65 °C

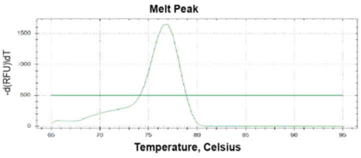

c-Myc B

Fw 5'-TGGGACATCTGAAATG-3'
Rv 5'-AAGGGTACATATTAGGACAACAGTCA TTC-3'
Tm 60 °C

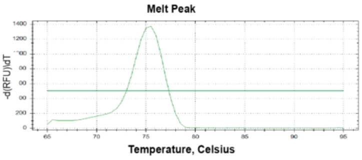

ABCC1

Melt peak

NF-κB

Fw 5'-CCCGAGTAGCTCGGATTACA-3'
Rv 5'- GGAGATTGAGACCAGCCTGA-3'
Tm 65 °C

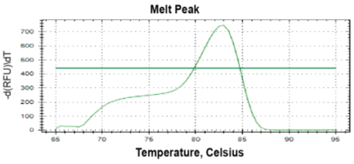

c-Myc A

Fw 5'-ACCGCTCTGGGTACGTG-3'
Rv 5'-GACCCGACCCTCCAAAAC-3'
Tm 60 °C

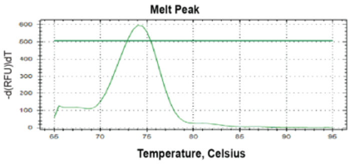

c-Myc B

Fw 5'-TGATGTGCCCTACCTGACCCT CGG-3'
Rv 5'-AGAGAGAAACAGTCGTGTCCA GATTTGCC-3'
Tm 66 °C

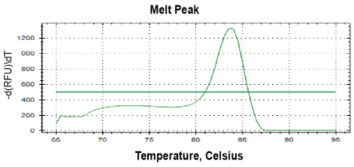

ABCC2

Melt peak

NF-κB A

Fw 5'-CCTGTTAGGTGGGACGTGAT-3'
Rv 5'- GGCAGAGGGGATAGGAAAAG-3'
Tm 65 °C

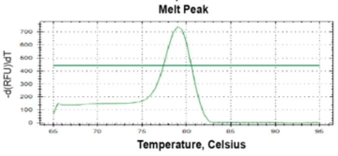

NF-κB B

Fw 5'-TATATGCATGCCTGGTTGTAGG-3'
Rv 5'- TCTCCAAGAACTATGGGACAAC-3'
Tm 61.6 °C

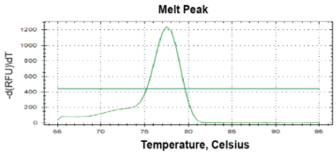

c-Myc A

Fw 5'-CCAAGGCAGAAGGATTGTTGAAGC-3'
Rv 5'-AACAGAGTGGTGGCATCAGTCG-3'
Tm 66 °C

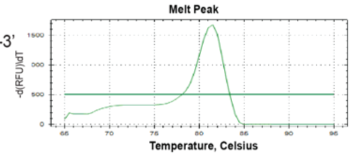

c-Myc B

Fw 5'-GTCTTCGTTCCAGACGCAGT-3'
Rv 5'-TTCTTGTTGGTGACCACCCT-3'
Tm 63 °C

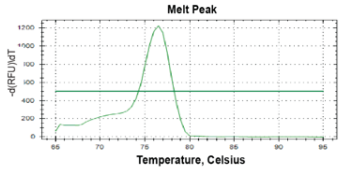

ABCC4

Melt peak

NF-κB

Fw 5'-ACAATATGGGTAATATCTCCCAGG-3'
Rv 5'- CACCATTGTGTACATGCTGTGA-3'
Tm 67.6 °C

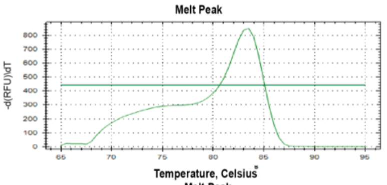

c-Myc

Fw 5'-CCACTGTTATCTGTTAGGCTTTA GGG-3'
Rv 5'-CTTCTCAGGACCAAACGACGG-3'
Tm 63 °C

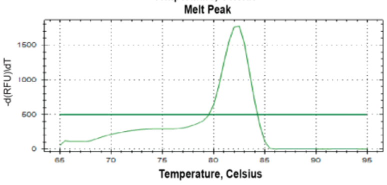

ABCC5

Melt peak

NF-κB A

Fw 5'-GGTGGATGGATCACAAGGTC-3'
Rv 5'- CTCTGTCCCTCAGGTTGGAGT-3'
Tm 65 °C

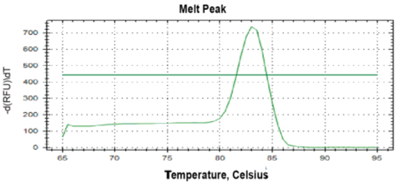

NF-κB B

Fw 5'-CGGTAGCTCACTCCTGTAATCC-3
Rv 5'- GATCTCGGCTCACTGTAACCTC-3
Tm 60 °C

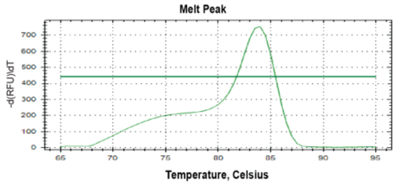

ABCC6

Melt peak

NF-κB A

Fw 5'-ACAATATGGGTAATATCTCCCAGG-3'
Rv 5'- CACCATTGTGTACATGCTGTGA-3'
Tm 65 °C

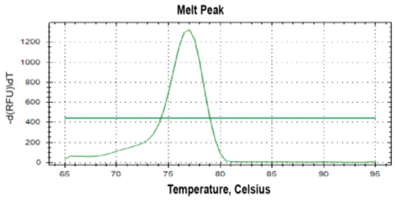

NF-κB B

Fw 5'-GGGGCAATATCTCCACAGAA-3'
Rv 5'- GCTGTGACCTCCCAGAAAGATA-3'
Tm 60 °C

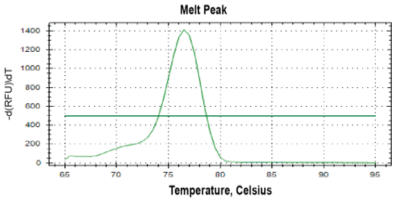

ABCG2

Melt peak

NF-κB

Fw 5'-GCCCGGCTAATTTTTGTATTTT-3'
Rv 5'- GGCACTACAGGAGGAGACTGA-3
Tm 60 °C

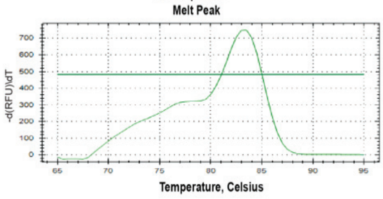

c-Myc

Fw 5'-GTAGTTAATCACTCTGGTTCATT CCGTTCG-3'
Rv 5'-TGCGGCTGGAGGTCACGATGG-3
Tm 66 °C

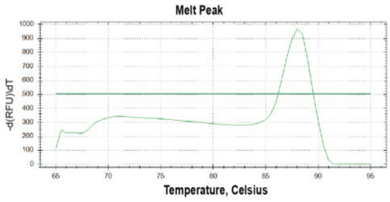

A

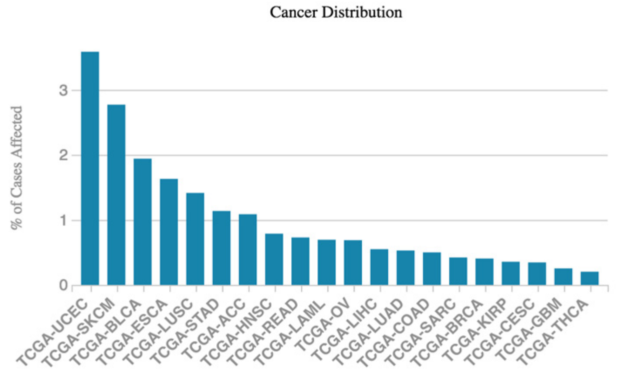
Cancer Distribution
% of Cases Affected
0
1
2
3
TCGA-UCEC
TCGA-SKCM
TCGA-BLCA
TCGA-ESCA
TCGA-LUSC
TCGA-STAD
TCGA-ACC
TCGA-HNSC
TCGA-READ
TCGA-LAML
TCGA-OV
TCGA-LIHC
TCGA-LUAD
TCGA-COAD
TCGA-SARC
TCGA-BRCA
TCGA-KIRP
TCGA-CESC
TCGA-GBM
TCGA-THCA

B

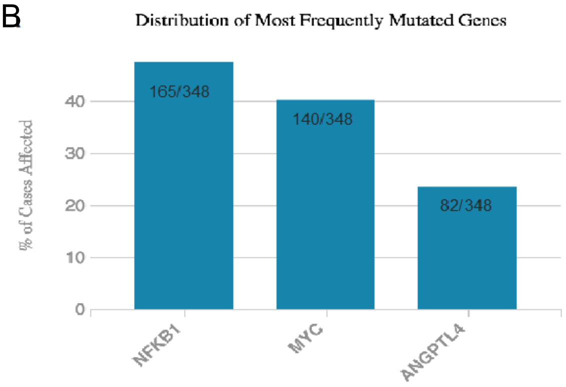
Distribution of Most Frequently Mutated Genes
% of Cases Affected
0
10
20
30
40
165/348
140/348
82/348
NFKB1
MYC
ANGPTL4

C

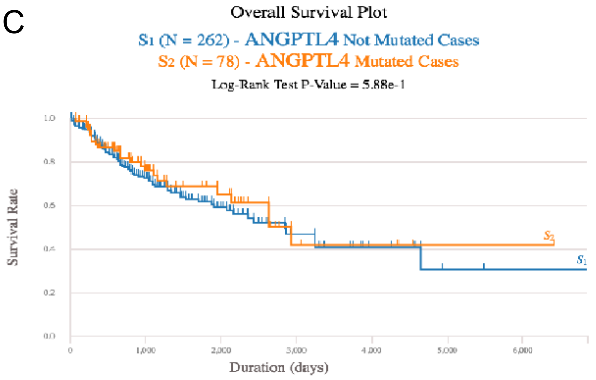
Overall Survival Plot
S1 (N = 262) - ANGPTL4 Not Mutated Cases
S2 (N = 78) - ANGPTL4 Mutated Cases
Log-Rank Test P-Value = 5.88e-1
Survival Rate
Duration (days)
S2
S1
